# Efficacy of fluralaner (Bravecto™ chewable tablets) for the treatment of naturally acquired *Linognathus setosus* infestations on dogs

**DOI:** 10.1186/s13071-017-2344-9

**Published:** 2017-09-18

**Authors:** Heike Kohler-Aanesen, Seppo Saari, Rob Armstrong, Karine Péré, Janina Taenzler, Eva Zschiesche, Anja R Heckeroth

**Affiliations:** 1 MSD Animal Health Norge AS, Thormøhlensgate 55, 5008 Bergen, Norway; 2 MSD Animal Health, Keilaranta 3, 02150 Espoo, Finland; 3 Merck Animal Health, 2 Giralda Farms, Madison, NJ 07940-1026 USA; 4 MSD Animal Health Innovation SAS, 7, rue Olivier de Serres, Angers Technopole CS67131, 49071 Beaucouzé, France; 5 MSD Animal Health Innovation GmbH, Research Antiparasitics, Zur Propstei, 55270 Schwabenheim, Germany

**Keywords:** Bravecto™, Chewable tablets, Dog, Efficacy, Exspot®, Fluralaner, Lice, Oral, Permethrin, *Linognathus setosus*, Topical

## Abstract

**Background:**

The clinical efficacy of fluralaner chewable tablets (Bravecto™, MSD Animal Health) against naturally acquired *Linognathus setosus* infestations on dogs was evaluated compared with permethrin (Exspot®, MSD Animal Health) treatment.

**Methods:**

Privately-owned dogs naturally infested with *L. setosus* from 21 different households were randomly allocated to two treatment groups. Fourteen dogs were treated once orally with fluralaner and ten dogs were treated once topically with permethrin, at the recommended label dose. Live *L. setosus* on all dogs were counted before treatment and 1, 7, 28 (both groups) and 84 (fluralaner group) days post-treatment according to a coat parting technique at pre-specified locations and lice species were confirmed microscopically. At the same time points, a veterinary dermatology severity score and an owner’s perceived pruritus score were recorded.

**Results:**

Percentage reduction in geometric mean *L. setosus* counts, comparing post- with pre-treatment counts within each group, were 85.7% (day 1), 96.8% (day 7) and 100% (days 28 and 84) for the fluralaner (two-sided two-sample *t*-test, *P* ≤ 0.0088 for days 1–84) and 67.5% (day 1), 90.3% (day 7) 99.1% (day 28) for the permethrin group (two-sided two-sample *t*-test, *P* ≤ 0.0014 for days 7–28). No lice were seen on fluralaner-treated dogs 28 and 84 days post-treatment. In contrast, two permethrin-treated dogs were re-treated at 7 and 28 days after initial treatment because of observed lice. Owner’s perceived pruritus scores were reduced compared to pre-treatment levels by 23.8% (day 1), 31.1% (day 7), 70.4% (day 28) and 99.5% (day 84) after fluralaner treatment and 21.3% (day 1), 45.8% (day 7), and 78.1% (day 28) after permethrin treatment. Dermatological signs were improved compared to pre-treatment levels in both treatment groups.

**Conclusions:**

Single oral fluralaner treatment eliminated natural *L. setosus* infestation on dogs within 28 days and led to complete dermatological recovery that was maintained until the study end on day 84. Single topical permethrin treatment reduced the number of *L. setosus* by 99.1% at day 28 although two animals required unscheduled re-treatment.

## Background

Lice are an occasional ectoparasite of dogs worldwide, but appear to be particularly common in colder climates including in the Scandinavian countries [1]. Lice are often the most common ectoparasites in these countries where the climate is less hospitable for fleas and ticks and, thus flea and tick parasiticides are not routinely used. In other geographical areas lice infestations occur sporadically, typically on dogs held in outdoor kennels [2].

Lice are highly host-specific ectoparasites and differentiated between biting lice, feeding on epidermal tissue debris and sebaceous secretions, and sucking lice, feeding on blood [3]. They spend their entire life on one host, and several lice species prefer specific locations on the host’s body. Predilection sites for lice are the head including ears, neck and back of the dog [4, 5]. Lice are usually unable to survive off the host animal for more than 2 days [3, 4]. Thus, lice are mainly spread by direct physical contact between individuals; contaminated brushes or combs can also transmit these parasites [3].

**Fig. 1:**
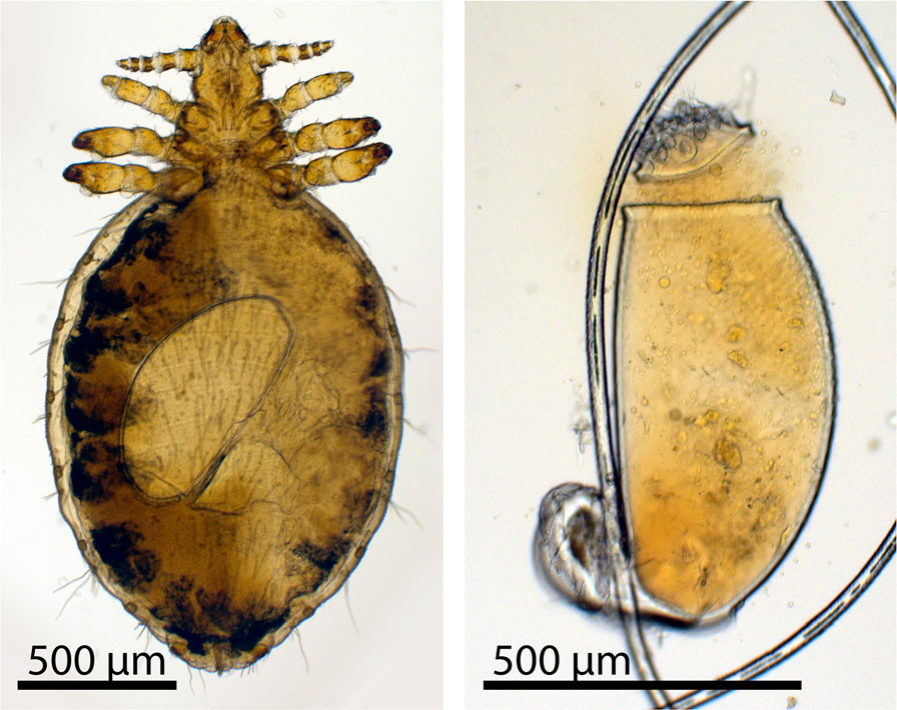
*Linognathus setosus* adult and nit under light microscope

The most common louse species causing canine pediculosis are the sucking louse *Linognathus setosus* and the chewing or biting louse *Trichodectes canis* [6–8]. Both species are flattened dorsoventrally and are easy to diagnose and differentiate, as the adults and their eggs (so-called “nits”) are visible to the naked eye [4]. The biting lice feed on skin scurf and other organic material on the skin. In contrast, the sucking lice feed on blood. Sucking lice are generally larger than biting lice and are grey to dusk red, depending upon the quantity of blood in their intestines. The head of sucking lice is narrower than the thorax and has elongated protrusible piercing mouthparts (Fig. 1). Biting lice have blunt heads that are wider than the thorax, are often of yellow color and move rapidly, while sucking lice move more slowly and may be seen head down close to the skin surface or actually feeding [7]. In Sweden and Finland the sucking louse *L. setosus* is the most prevalent louse species leading to heavy infestations usually seen during the winter months [6–8].

Development of *L. setosus* from egg to adult takes 3 to 6 weeks and occurs only on the host, where all stages are able to suck blood [3, 9–11]. By having pincer-like tarsal claws, adult lice cling to the host hair shafts, usually near the base [2, 10]. Females lay 50 to 100 eggs (nits), about one egg every 1 or 2 days, which are securely attached to the hairs by insoluble cement [2, 3, 9]. The nymph hatches after 1 to 2 weeks and within another 1 to 3 weeks molts through three to five stages to develop into an adult louse (imago) that will live on its host for around 30 days [3].

Lice are highly active and heavy infestations can cause severe skin irritation and debilitation with erythema, scaling, crusting, papules and pruritus. The heavy itching (pruritus) and associated scratching leads to traumatic skin damages including alopecia and secondary bacterial infection (pyoderma). Affected animals are often restless [4, 10, 11]. However, even heavy infestation can be clinically innocuous, while in some dogs the mild infestation leads to relentless itching through hypersensitivity reaction. Anemia is possible, especially in young or immune-suppressed animals, caused by blood loss due to heavy *L. setosus* infestations [4, 10].

Several insecticides including permethrin, fipronil and imidacloprid as well as macrocyclic lactones such as selamectin are reported to be effective against *L. setosus* infestations [1, 4, 12]. Fluralaner is a novel isoxazoline insecticide and acaricide that provides immediate and persistent efficacy against fleas and ticks for 12 weeks [13]. A single fluralaner administration to dogs is highly effective against generalized demodicosis [14] and infestations with *Sarcoptes scabiei* spp. and *Otodectes cynotis* mites [15, 16]. The present field study evaluated the clinical efficacy of a single oral fluralaner administration (Bravecto™ chewable tablets, MSD Animal Health) against naturally acquired *L. setosus* infestations on dogs in Norway. Permethrin (Exspot®, MSD Animal Health) is registered in Norway as a topically applied treatment against *L. setosus* on dogs, and was administered as a positive control. The effectiveness of fluralaner was evaluated for the registered 84 days and for permethrin for the registered 28 days effectiveness after single treatment.

## Methods

### Study set-up

The study was a multicenter, randomized, positive controlled, field efficacy study, conducted between September 2015 and July 2016 with the assistance of nine veterinary practices in Norway, which diagnosed lice infestations in privately-owned dogs of any breed and gender. Blinding of the study was assured through the separation of study functions. Veterinarians performing dermatological evaluations and lice counts and animal owners performing pruritus assessments post-treatment were masked regarding the treatment allocation of dogs by having a third person administering the treatment.

### Inclusion criteria

Natural *L. setosus* infestation, on otherwise healthy dogs on initial physical examination, was confirmed by visual coat examination using the hair coat parting technique and microscopic diagnosis of the louse species. One louse present was sufficient for study inclusion. Dogs with visible lice-associated dermatological signs, e.g. erythema, scaling, crusting, alopecia, and/or secondary pyoderma, were included in the study with the owner’s informed consent. Dogs were excluded if they were treated with any ectoparasitic product within the previous 30 days or with short or long acting glucocorticoids within the previous 7 or 30 days, respectively. Dogs were also excluded if they showed evidence of other systemic disease, had evidence of allergic dermatitis, had not lived continuously at the owner’s household for the previous 2 months, or lived in a household with more than two other dogs. All dogs in one household were administered the same treatment, but only dogs meeting the inclusion criteria were included in further observations, assessments and efficacy calculations.

During the study, each dog remained with its owner in its usual housing and diet. Contact with other animals was not restricted during the study. Dogs fulfilling the inclusion criteria were allocated by household in which the dog lived in blocks of two to one of the two study groups, using a veterinarian specific, computer generated randomization list.

### Details of dogs included

Twenty-four dogs (8 female, 16 male) between 9 months and 13 years old, weighing between 5.0 and 47.6 kg on the day of treatment and living in 21 households were included in the study. Three mixed breed dogs and 21 purebred dogs were enrolled. Besides various small and large as well as short and long haired breeds, the following FCI (Federation Cynologique Internationale) group standards were represented: Sheepdogs and Cattledogs (except Swiss Cattledogs) (*n* = 4); Pinscher and Schnauzer - Molossoid and Swiss Mountain and Cattledogs (*n* = 3); Spitz and Primitive Types (*n* = 3); Pointers and Setters (*n* = 1); Retrievers, Flushing Dogs, Water Dogs (*n* = 7); Companion and Toy Dogs (*n* = 3).

### Treatment

One group of dogs (*n* = 14) was treated once orally with fluralaner chewable tablets (Bravecto™) based on individual body weight according to label recommendations. Another group of dogs (*n* = 10) was treated once topically at one to three sites on the back with permethrin (Exspot®) according to label recommendations. Fluralaner-treated dogs also received a topical application of propylene glycol equivalent to the permethrin volume they would have received if assigned to the permethrin group. This application was designed to ensure that personnel performing skin and lice assessment were masked to the dogs’ treatment group. Following treatment, dogs in both treatment groups were not allowed to be bathed or water-immersed for at least 7 days.

### Lice assessments and clinical findings

*Linognathus setosus* on all dogs were counted before treatment (initial examination) and 1, 7, 28 (both groups) and 84 (fluralaner group) days post-treatment. On each occasion the number of live nymphs and adult lice were counted with the naked eye by the veterinarian in approximately 5 cm long coat partings at 25 predetermined locations including the predilection sites of the shoulder, neck and back according to the coat parting technique described by Pollmeier et al. [17]. Additionally, on the initial examination the diagnosis of an infestation with *L. setosus* was confirmed under a light microscope.

At the same time points, a veterinarian dermatology severity score and an owner’s perceived pruritus score were recorded. Each dog’s clinical signs and the extent of its lice-associated dermatological lesions were assessed by the veterinarian and classified as: 0, healthy skin with no evidence of any abnormality; 1, mild dermatological lesions with erythema, scaling and crusting; 2, moderate dermatological lesions with changes due to secondary to pruritus (pyoderma and focal alopecia); and 3, severe dermatological lesions including severe self-trauma, secondary pyoderma and multifocal alopecia. The owner was asked to assess the dog’s pruritus using a Pruritus Visual Analogue Scale (PVAS) chart according to Olivry et al. [18]. Briefly, the owner matched their perception of the pruritus signs displayed by the dog with a severity scale on the PVAS chart. The PVAS score was then calculated as the distance from the bottom of the chart to the owner’s mark as a percentage of the total length of the chart.

### Data analysis

The statistical analysis was performed using the software package SAS® (SAS Institute Inc., Cary, NC, USA, release 9.4) with the individual dog as experimental unit. The primary assessment variable in each group was the total number of live lice (nymphs and adults) at each assessment time point post-treatment compared to pre-treatment counts. The percentage efficacy against *L. setosus* lice was calculated using geometric means emplyoing Abbott’s formula:

$$ \mathrm{Efficacy}\kern0.5em \left(\%\right)\kern0.5em =\kern0.5em 100\times \left({\overline{x}}_{pre- treatment}\hbox{--} {\overline{x}}_{post- treatment}\right)/{\overline{x}}_{pre- treatment}, $$

where ${\overline{x}}_{pre- treatment}$ is the mean number of total live lice counts pre-treatment, and ${\overline{x}}_{post- treatment}$ is the mean number of total live lice counts at each assessment time point post-treatment.

To allow the calculation in case of zero counts, the geometric mean was calculated as follows:

$$ {\mathrm{x}}_{\mathrm{g}}={\left(\prod_{\mathrm{i}=1}^{\mathrm{n}}{\mathrm{x}}_{\mathrm{i}}+1\right)}^{\frac{1}{\mathrm{n}}}\hbox{--} 1 $$

where n is the number of animals, i-th animal and x_i_ the number of lice on the i-th animal. Log-transformed [ln(x + 1)] counts of *L. setosus* lice were used to confirm the efficacy calculation.

Lice counts post-treatment were compared pairwise to lice counts pre-treatment using a two-sided two-sample *t*-test (α = 0.05). To compensate for the skewed distribution, the lice counts were log-transformed and shifted prior to analysis using the x_i_’ = ln(x_i_ + 1) transformation.

The veterinarian dermatology severity and owner’s perceived pruritus scores pre- and post-treatment were compared for each study group and assessment time point using Wilcoxon’s signed rank test (one-sided, α = 0.025).

The percentage reduction for the owner’s perceived pruritus score was calculated using arithmetic means emplyoing Abbott’s formula:

$$ \mathrm{Reduction}\kern0.5em \left(\%\right)\kern0.5em =\kern0.5em 100\times \left({\overline{x}}_{pre- treatment}\hbox{--} {\overline{x}}_{post- treatment}\right)/{\overline{x}}_{pre- treatment}, $$

where ${\overline{x}}_{pre- treatment}$ is the mean score pre-treatment, and ${\overline{x}}_{post- treatment}$ is the mean score at each assessment time point post-treatment.

The percentage of lice-free dogs and lice-free households for each study group and assessment time point was calculated as follows: number of dogs or households free of lice divided by the total number of dogs or households in the group. For day 1, day 7 and day 28 post-treatment, non-inferiority of the percentage of parasite free dogs in the fluralaner-treated group was compared to the permethrin- treated group using the Farrington-Manning test of non-inferiority with a level of significance of α = 0.025 and a tolerated difference of δ = 0.15 [19]. Both, *P*-value and lower 97.5% one-sided confidence limit were calculated. If the lower confidence limit was above −0.15, non-inferiority was concluded and if the lower confidence limit was above 0, superiority was concluded.

## Results

No adverse event related to fluralaner or permethrin administration was observed in any dog. Single oral fluralaner treatment eliminated natural *L. setosus* infestation on dogs within 28 days post-treatment (100% efficacy). Permethrin administered once topically reduced the number of *L. setosus* by 99.1% at 28 days post-treatment. This reduction in lice counts was significant for both treatment groups (*P* < 0.0001) (Table 1). No lice were seen in fluralaner-treated dogs at 84 days post-treatment (100% efficacy; *P* < 0.0001). In contrast, two permethrin-treated dogs were observed to have lice and were re-treated with an alternative anti-lice product. One dog was withdrawn from the study in the first week after treatment because the owner observed lice and treated with an alternative lice treatment. Another dog was observed to have many nits and a live louse at 28 days post-treatment, and was treated with an alternative lice treatment.

**Table 1:** Mean lice counts and efficacy (%) after a single oral administration of fluralaner or topical administration of permethrin to dogs naturally infested with lice *L. setosus*

	Study day	Dogs^a^	Count range (*n*)	Geometric mean lice count	Efficacy (%)	*P*-value^c^
Fluralaner	0	14	1–115	8.0	–	–
	1	14	0–38	1.6	85.7	0.0088
	7	14	0–3	0.3	96.8	< 0.0001
	28	13	0–0	0.0	100	< 0.0001
	84	11	0–0	0.0	100	< 0.0001
Permethrin	0	10	1–36	10.2	–	–
	1	10	0–25	3.3	67.5	0.0854
	7	9^b^	0–7	1.0	90.3	0.0014
	28	8^b^	0–1	0.1	99.1	< 0.0001

^a^Number of dogs presented to visit

^b^Dog withdrawn from the study due to insufficient permethrin treatment effect against *L. setosus*

^c^Two-sided two-sample *t*-test (α = 0.05)

Fluralaner treatment eliminated lice from treated dogs by 28 days post-treatment and all fluralaner-treated dogs remained lice-free at 84 days post-treatment. Permethrin treatment also increased the percentage of lice-free dogs but did not clear all dogs of lice at any time point (Table 2). A similar result was seen in the percentage of lice-free households with fluralaner achieving 100% at 28 and 84 days post-treatment and permethrin reaching 83% at 28 days post-treatment. Fluralaner was significantly superior to permethrin for both lice-free dogs and lice-free households at 1 day post-treatment. At 7 and 28 days post-treatment fluralaner was significantly non-inferior to permethrin for both lice-free dogs and lice-free households (Farrington-Manning test of non-inferiority for the risk difference, dogs: *P =* 0.0070 and *P* = 0.177; households: *P* = 0.0121 and *P* = 0.0147). treated with an alternative lice treatment. Another dog was observed to have many nits and a live louse at 28 days post-treatment, and was treated with an alternative lice treatment.

**Table 2:** Lice-free dogs and households after a single oral administration of fluralaner or topical administration of permethrin to dogs naturally infested with lice *L. setosus*

Study day	Lice-free dogs				Lice-free households			
	Fluralaner (%)	Permethrin (%)	Lower 97.5% CL^a^	*P*-value^b^	Fluralaner (%)	Permethrin (%)	Lower 97.5% CL^a^	*P*-value^b^
1	64	20.0	0.0417	0.0019	62	13	0.0554	0.0020
7	79	44.4	-0.0502	0.0070	77	43	-0.0858	0.0121
28	100	87.5	-0.1311	0.0177	100	83	-0.1182	0.0147
84	100	na	–	–	100	na	–	–

*Abbreviation*: *na* not applicable

^a^Confidence limits based upon the Farrington-Manning test of non-inferiority for the risk difference. Results > -0.15 for significant non-inferiority and results > 0 for significant one-sided superiority with α = 0.025

^b^Farrington-Manning test of non-inferiority for the risk difference

**Fig. 2:**
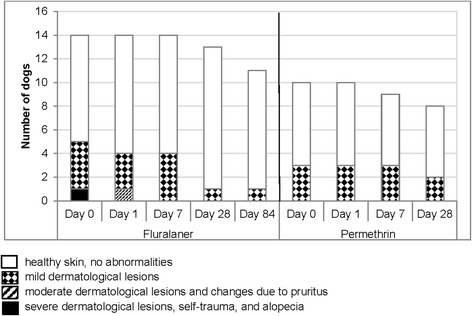
Results of the veterinary dermatology severity score

**Fig. 3:**
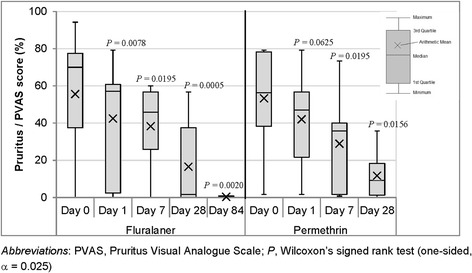
Results of the owner’s perceived pruritus score

Of the 14 fluralaner-treated dogs, 12 dogs showed up for all five post-treatment study visits and of the 10 permethrin-treated dogs eight dogs showed up for all four post-treatment visits (Table 1). One permethrin-treated dog was withdrawn as described above. The veterinary dermatology severity scores decreased following fluralaner and permethrin treatment although the difference was not significant in either study group (Fig. 2). Before treatment, the mean owner’s perceived pruritus score was comparable in both study groups (fluralaner group: 55.6%, permethrin group: 53.3%). After treatment it decreased significantly in both groups (Fig. 3).

## Discussion

Fluralaner administered once orally completely eliminated naturally acquired *L. setosus* infestation from treated dogs by 28 days post-treatment and dogs remained lice-free on re-examination until the end of the 12-week assessment period. The *L. setosus* life-cycle takes place only on the host and lasts up to 6 weeks, thus treatment needs to remain effective for at least 6 weeks to eliminate newly hatched lice. Permethrin was highly effective, but did not lead to a completely lice-free population at 4 weeks and further re-treatment would be required to eliminate lice from the household.

Dogs in both groups also showed improved veterinary dermatology severity scores and owner’s perceived pruritus scores consistent with the reductions in lice counts. The improvements in the veterinarian-assessed dermatology severity scores after fluralaner or permethrin treatment were not significant; however, a substantial portion of the lice infested dogs were assessed as having healthy skin before treatment administration (Fig. 2). The improvement in the owner’s perceived pruritus scores was significant in both groups showing that the reduced perception of pruritus is associated with successful lice treatment. Reduced perception of pruritus can lead to misinterpretation of treatment success by the owner, as even the presence of one louse is sufficient for remission of an *L. setosus* infestation. Thus, a single treatment is not always sufficient for complete control of lice on dogs.

Only few studies are reported regarding treatment of *L. setosus* infestation in dogs. A field study in Norway in 27 dogs with natural *T. canis* or *L. setosus* lice infestations using imidacloprid and moxidectin (Advantage®, Bayer Animal Health) applied once topically reported a knock down effect against both lice species within 24 h with no reported recurrence of lice over the 6 week study period [1]. An investigation of selamectin or permethrin efficacy against *Linognathus* in 21 naturally infested dogs in Sweden found that at 7 days post-treatment, 11 selamectin treated dogs were lice-free, while lice were found on three of ten permethrin-treated dogs [4]. This study did not examine the dogs subsequent to the 7 days to determine, if there was recurrence in either treated group.

The results of this field study clearly demonstrate the insecticidal efficacy of fluralaner against natural infestations with *L. setosus* in dogs. Due to its systemic activity and 84 days treatment interval, the duration of efficacy exceeds the egg-to-egg development of *L. setosus* and inhibits a remission of lice infestation during this time period.

A concern with use of permethrin for treating lice infested dogs is the risk of neurotoxic signs in cohabiting cats, possibly even leading to mortality [20]. This concern may inhibit the willingness of owners to retreat dogs in this situation and may be a factor leading to treatment failure. There are no toxicity concerns with use of fluralaner in households with cats, and there are fluralaner presentations approved for treating cats [13].

## Conclusion

Single oral administration of fluralaner to naturally infested dogs completely eliminated *L. setosus* within 28 days. Topical permethrin treatment is also highly effective, but a single treatment did not always completely eliminate lice and re-treatment is likely to be required. Fluralaner treatment remarkably improves dermatological signs over a 12 week observation period.
